# Antianxiety-Like Effects of Chimpi (Dried Citrus Peels) in the Elevated Open-Platform Test

**DOI:** 10.3390/molecules180810014

**Published:** 2013-08-20

**Authors:** Aya Ito, Noriyuki Shin, Takashi Tsuchida, Toshiki Okubo, Hisayoshi Norimoto

**Affiliations:** Kampo Research Laboratories, Kracie Pharma Ltd., Kanebo machi 3-1, Takaoka City, Toyama 933-0856, Japan; E-Mails: ito_aya@phm.kracie.co.jp (A.I.); shin_noriyuki@phm.kracie.co.jp (N.S.); tsuchida@phm.kracie.co.jp (T.T.); t_okubo@phm.kracie.co.jp (T.O.)

**Keywords:** Dried citrus peels (Chimpi), antianxiety-like effects, hesperidin, hesperetin, yokukansankachimpihange, elevated open-platform test

## Abstract

Dried citrus peels (Chimpi) is one of the most common natural medicines with *qi* (energy flow) rectifying and *shi* (dampness) drying actions, which originates from *Citrus unshiu*, and/or *C. reticulata* according to the definition of the pharmacopoeiae of Japan and China. In this study, the pharmacological effects of their extracts and major chemical constituents hesperidin and its aglycone hesperetin on anxiety were examined with an anxiety model of elevated open-platform test using ICR male mice (6-week-old) and total duration of freezing was decreased in fluoxetine-treated mice, which is a simple and highly sensitive to the effects of serotonergic anxiolytics. Moreover, yokukansankachimpihange (YKH), a combination of yokukansan with Chimpi and Hange (Pinellia) was also examined because Chimpi is considered to play a crucial part in this formula against anxious symptoms in dementia patients. The results showed that Chimpi and YKH possess a significant anxiolytic-like effect similar to that of fluoxetine, suggesting that they might be similar to fluoxetine in their pharmacological actions through the serotonergic neurotransmission pathway. Moreover, it also suggested that the major chemical constituent, hesperidin could be an active principle attributed to the antianxiety-like effects with a direct and indirect role via its aglycone hesperetin.

## 1. Introduction

Chimpi (Chenpi in Chinese) is one of the most common natural medicines with *qi* (energy flow) rectifying and *shi* (dampness) drying actions, which originates from the dried peels of ripe fruit of *Citrus unshiu* Marcowicz, and/or *C. reticulata* Blanco according to the definition of the pharmacopoeiae of Japan and China [1,2]. 

The terms of *qi* rectifying and *shi* drying in traditional Chinese-Japanese medicine, are unique concepts in the treatment of immunologic, digestive and psychological disorders [3]. Therefore, Chimpi is applied mostly in the traditional Chinese-Japanese formulae such as hochuekkito [4,5], rikkunshito [6] and yokukansankachimpihange (YKH) [7,8] for improving immunologic functions, anorexia and psychological disorders. However, the pharmacological study on Chimpi still has not been studied adequately except for several reports describing in immune system modulating activity [9] and gastrointestinal mobility [10]. 

Recently, owing to the fact that Yamakuni *et al.* found that nobiletin, a polymethoxylated citrus flavone showed activity against dementia in an animal study, Chimpi is expected to be a promising functional food for the prevention of dementia [11]. Furthermore, more attention has been paid to the formula YKH, a combination of yokukansan with Chimpi and Hange (Pinellia) for treatment of behavioral and psychiatric symptoms of dementia (BPSD) such as physical aggression, agitation, anxiety and depressive mood [12,13] since the suggestion by Yamakuni’s study group. In addition, YKH is indicated for adults (especially those past middle age) with obvious neuropathy as Chimpi and Hange (Pinellia) are added, not like yokukansan used originally for treating restlessness, agitation and night crying in children [14]. These facts suggest that Chimpi may play a crucial role in their psychopharmacological actions. 

Chimpi is characterized by the presence of abundant flavonoids which have been known to possess a variety of activities against inflammation, oxidation, cholesterol synthesis and so forth. Among them, hesperidin, which, unlike nobiletin, is abundant in citrus fruits including in Chimpi, and known to be as a chemical marker for quality control of Chimpi materials [1,2] was reported to have neuroprotective effects in a recent study [15]. 

Thus, this study was mainly focused on the pharmacological effects of Chimpi (extracts of *C. unshiu* and *C. reticulata*) and hesperidin, mainly present in Chimpi, and its aglycone hesperetin on anxiety in a novel animal anxiety model, elevated open-platform test. Namely, mice are placed on an elevated open-platform which induces a psychological stressor without a physical and/or painful stimulus, and the mice display typically freezing behaviors used as an index of anxiety [16]. Besides, study on the antianxiety-like effects of YKH is also performed in the same model.

## 2. Results and Discussion

### 2.1. HPLC Analysis

**Figure 1:**
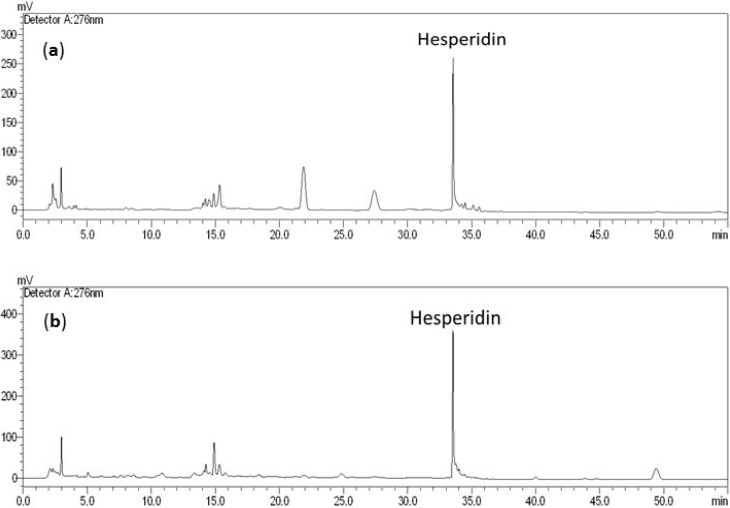
(**a**) HPLC profile of CU-ext (from *Citrus unshiu*). (**b**) HPLC profile of CR- ext (from *C. reticulata*). Contents of hesperidin was calculated on the basis of a standard curve (y = 16,882x + 5,460.2, *r*^2^ = 1) with concentration range (0.244~500 μg/mL).

Hesperidin has been designated as a chemical maker for the quality control of Chimpi in the Chinese and Japanese pharmacopoeiae [1,2], therefore, HPLC analysis of both CU- and CR-extract from *Citrus unshiu* and *C. reticulata* were carried out before starting their pharmacological examinations. As shown in Figure 1, both of CU- and CR-extract showed a similar HPLC profile indicating hesperidin (Rt: 33.6 min) was the major chemical constituent. Its contents in CU-extract (127.0 mg/g) were more than in that of CR-extract (184.0 mg/g), in the light of absolute quantitative chemical analysis. Meanwhile, hesperetin (Rt: 41.7 min), an aglycone of hesperidin was not detected in either of them.

### 2.2. Effects on Anxiety-Like Behavior in the Elevated Open-Platform Test and Locomotor Activity

**Figure 2:**
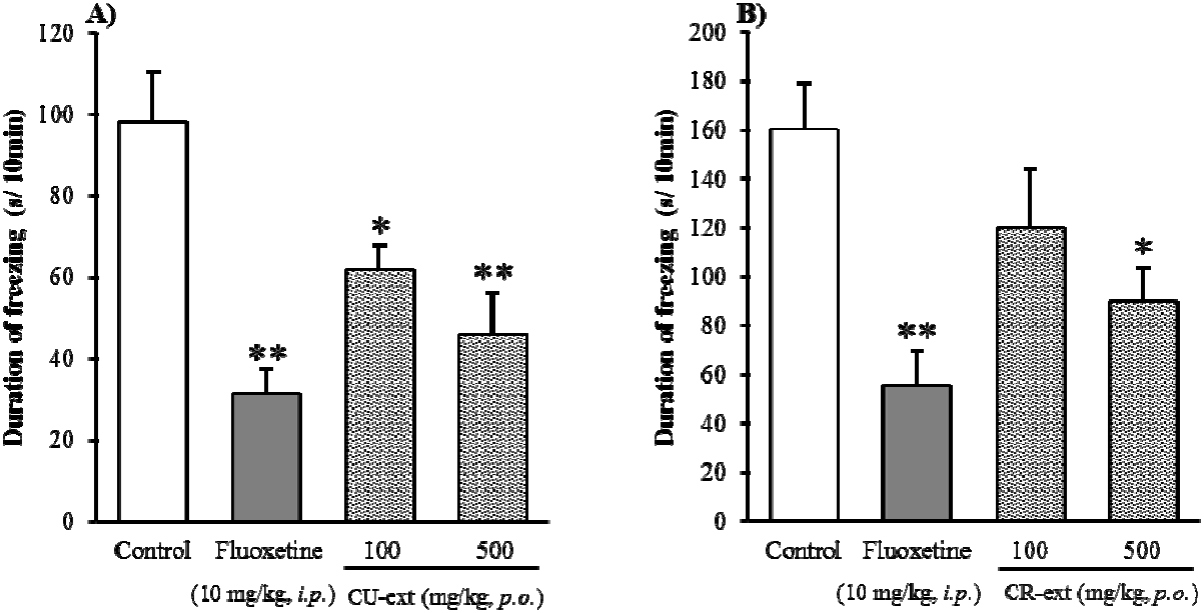
Effect of Chimpi extract (CU- and CR-ext) on the duration of freezing in the elevated open-platform test. Each column represents the mean ± S.E. of eight mice. * *P* < 0.05, ** *P* < 0.01 *vs*. Control (one-way ANOVA followed by Dunnett’s test).

When the mouse was placed on elevated open-platform, behavioral responses to the stressor were observed, and a freezing behavior (a state without movements except for respiration) was regarded as an anxiety-like behavior [16]. In the light of results of a pre-test, fluoxetine (0.1, 1.0, 10 mg/kg, i.p.) decreased the duration of freezing in mice in a dose-dependent manner (data not shown), particularly at dose of 10 mg/kg was significant, which is consistent with reports from Miyata *et al.* [13]. Thus, fluoxetine with a dose of 10 mg/kg was used in the following studies on Chimpi and YKH extracts, hesperidin and hesperetin. As shown in Figure 2A,B, both CU- and CR-ext from *C. unshiu* and *C. reticulata* dose-dependently and significantly decreased duration of freezing as compared with control. No significant differences were observed among CU-, CR-ext and the positive control fluoxetine.

**Figure 3:**
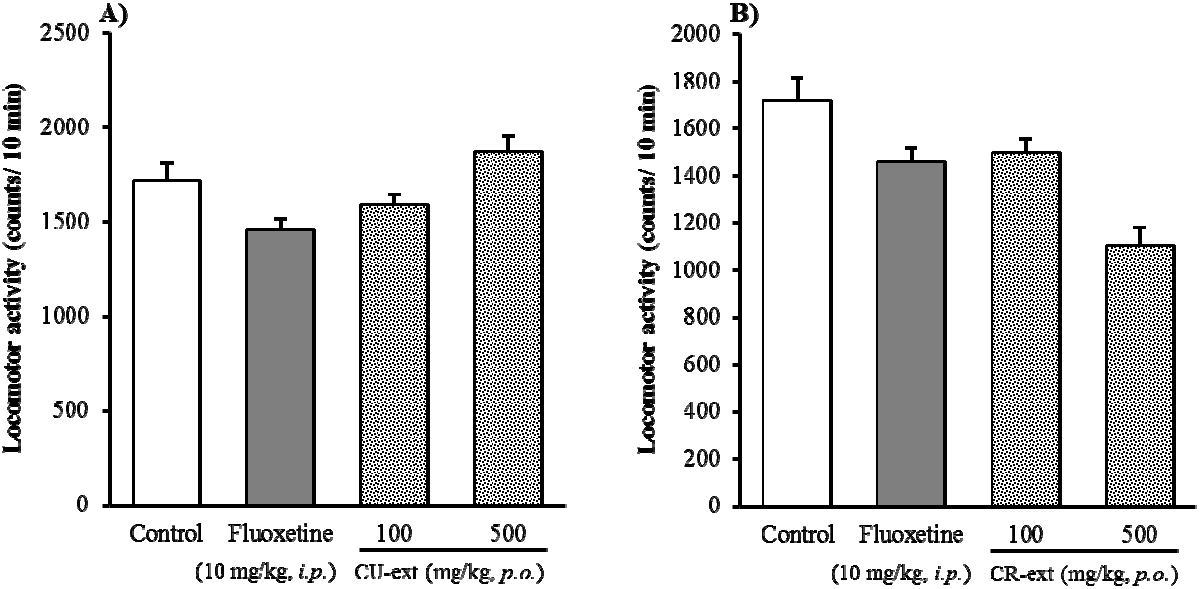
Effect of Chimpi extracts (CU- and CR-ext) on the locomotor activity. Each column represents the mean ± S.E. of eight mice (one-way ANOVA followed by Dunnett’s test).

Furthermore, the effects of both CU- and CR-ext on locomotor activity were examined (Figure 3), and they did not affect significantly locomotor activity of mice though a tendency to decreases observed as well as fluoxetine.

These results indicated that Chimpi both from *C. unshiu* and *C. reticulata* possessed antianxiety-like effects rather than sedative effects. Moreover, we also have examined the effect of these Chimpi extracts in the light/dark box test (data not shown), and found that they did not have antianxiety-like effects in this model, because light/dark box test is commonly used to predict or screen anti-anxiety drugs by characterizing the mechanism of action to γ-aminobutyric acid (GABA)/benzodiazepine neurotransmission [17]. Compared to the light/dark box test, the elevated open-platform test is a simple and useful anxiety model for detecting serotonergic anxiolytics [16]. Together, these findings furthermore suggested that the possible pharmacological action of Chimpi against anxiety-like effects might be similar to that of the positive control fluoxetine, a selective inhibitor of serotonin reuptake. In other words, we speculate that its pharmacological actions may be associated with serotonergic neurotransmission rather than the γ-aminobutyric acid (GABA)/benzodiazepam neurotransmission pathway, and further studies on this are ongoing.

**Figure 4:**
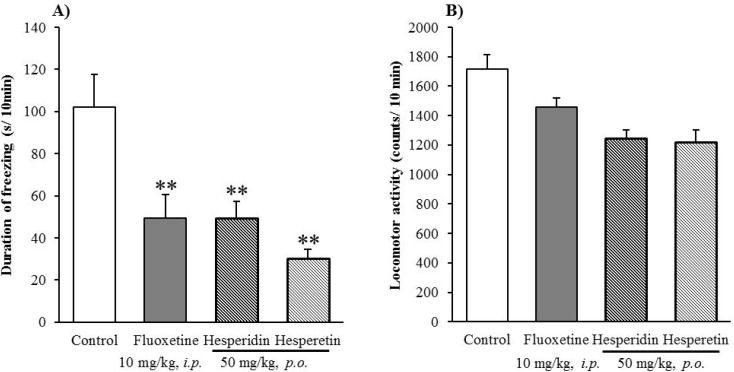
Effect of hesperidin and hesperetin on the duration of freezing in the elevated open-platform test (**A**) and locomotor activity (**B**). Each column represents the mean ± S.E. of eight mice. ** *P* < 0.01 *vs.* Control (one-way ANOVA followed by Dunnett’s test).

Moreover, the major chemical constituent hesperidin and its aglycone hesperetin contained in Chimpi were examined at a dose of 50 mg/kg (p.o.) in the same method, and it was found that they significantly decreased the duration of freezing in mice without significant effects on their locomotor activities (Figure 4A,B). Although hesperetin showed stronger antianxiety-like effects than hesperidin in the elevated open-platform test, there was no significant difference between them.

In general, flavonoids such as hesperidin are known to possess in *in vitro* studies various physiological activities such as antioxidant, anti-inflammation and neuroprotection effects [15,18,19], however, like many other flavonoids, hesperidin has poor bioavilability and understanding of their absorption, distribution and metabolism. Lee *et al.* reported that hesperidin is metabolized to hesperetin by intestinal bacteria [20], and hesperetin-7-*O*-β-D-glucuronide, an analogue of hesperidin (hesperetin-7-*O*-β-D-rutinoside) and hesperetin are able to traverse the blood-brain barrier (BBB) in *in vitro* studies [21]. In particular, hesperetin can traverse the BBB easier than its glycosides such as hesperetin-7-*O*-β-D-glucuronide and hesperetin-5-*O*-β-D-glucuronide. Moreover, hesperetin is more bioavailable than hesperidin, regardless of the route of administration in *in vivo* pharmacokinetic studies [22]. These facts suggest that hesperidin, as an active principle, could play a direct and/or indirect role in the antianxiety-like effects of Chimpi.

Dried citrus peels is prepared traditionally by a lengthy complex steaming and fermentation procedure and is designated as Chimpi, because Chimpi so processed is considered to have better quality and greater pharmacological actions than air- or sun-dried peels. Nevertheless, air- or sun-dried citrus peels are used in traditional Chinese-Japanese medicine, although the prior transformation of hesperidin to hesperetin via fermentation can alter the effects or pharmacokinetics of hesperidin [22]. Therefore, there is the problem that the crude drug or formulae with the same name may differ in their chemistry and/or pharmacology, because their specifications or processing methods may not be same in different countries such as in China and Japan.

**Figure 5:**
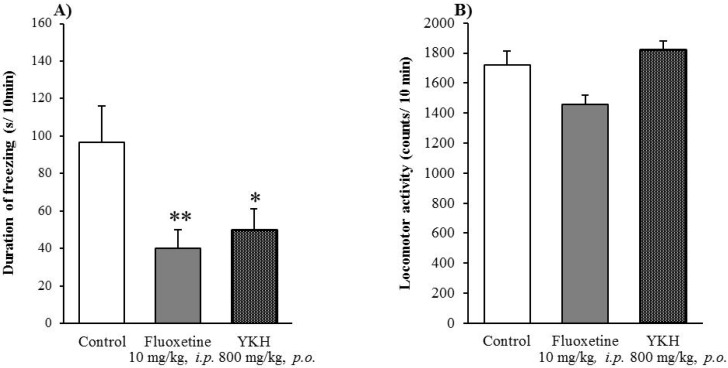
Effect of YKH on the duration of freezing in the elevated open-platform test (**A**) and locomotor activity (**B**). Each column represents the mean ± S.E. of eight mice. * *P* < 0.05, ** *P* < 0.01 *vs.* Control (one-way ANOVA followed by Dunnett’s test).

Similarly, YKH at a dose of 800 mg/kg significantly decreased the duration of freezing and did not affect locomotor activities of mice as well as fluoxetine in the elevated open-platform test and locomotor activity test, respectively (Figure 5A,B).

Based on the above results, Chimpi may play a role in the antianxiety-like effect of YKH, however, it is necessary to further examine and compare the effects of other crude drugs including Hange (Pinellia) whether they also contribute to the antianxiety-like effects. In addition, further studies to focus on dose-response effects and chemical analysis are needed. With respect of this point, a detailed pharmacological study and chemical analysis of active constituents are now in progress.

## 3. Experimental

### 3.1. Plant Material and Extract Preparation

*Citrus unshiu* Marcowicz (Lot No. B6103012) was purchased from Uchida Wakanyaku Ltd. (Tokyo, Japan), and *C. reticulata* Blanco (H120323210) was provided and identified by Prof. Ching-Chiung Wang (Institute of Pharmacognosy Science, Taipei Medical University, R.O.C). Each sample of dried peels (280 or 400 g) was pulverized and extracted with 1.5 or 2.0 L water under reflux for 30 min, and lyophilized to give extract with yields of 23.4% (CU-ext) and 14.6% (CR-ext). In addition, their voucher specimens are deposited at the herbarium of Kampo Research Laboratories, Kracie Pharma, Ltd with management numbers YL-C-001 and YL-C-002, respectively.

YKH extract was obtained according to the standard decoction preparation methods prescribed by the notification (1985) of the Japanese government Ministry of Health and Welfare. Briefly, YKH consisting of Pinelliae tuber (Lot No. 11614410; 5.0 g), Atractylodis rhizoma (Lot No. 10906210; 4.0 g), Poria (Lot No. 11523210; 4.0 g), Cnidii rhizoma (Lot No. 11N05520; 3.0 g), Citrus unshiu peels (Lot No. B610312; 3.0 g), Angelicae radix (Lot No. 11927110; 3.0 g), Bupleuri radix (Lot No 11620210; 2.0 g), Glycyrrhizae radix (Lot No. 11921520; 1.5 g), and Uncariae uncis cum ramulus (Lot No. 11214211; 3.0 g) was decocted with water (570 mL). When the water decoction was concentrated to half its volume, it was subjected to lyophilization to give a dried powder (5.4 g). Except for Citrus peels, other crude drugs were obtained from GMP-standardized plants from Kracie Pharma, Ltd.

### 3.2. HPLC Profiles of CU- and CR-Ext

HPLC profiles of both Chimpi extract CU- and CR-ext were obtained with a Shimadzu HPLC liquid chromatography system equipped with Lc-10ATVP and a reversed-phase column (Purospher^®^ STAR RP-18e, 4 mm i.d. × 250 mm, Merck, Darmstadt, Germany, Column temperature: 30 °C) and an SPD-10A UV-VIS detector at 276 nm. The column was equilibrated with solvent A (acetonitrile) and B (0.05% TFA), then the ratio of solvent A was increased to 10% in 10 min, 18% in 20 min, and 39% in 25 min with a flow rate of 1 mL/min.

### 3.3. Experimental Animals

Male ICR mice (5-weeks-old) were purchased from SLC, Inc. (Shizuoka, Japan). They were group-housed in a temperature-, humidity-controlled room on a 12-h light/dark cycle (light off, 8:00 PM) and provided laboratory pellet chow and water *ad libitum*. Before the experimental procedures, they were acclimated to the room for 1 week. This study was approved by Experimental Animal Care Committee of Kracie Pharma, Ltd (approval No. 100157, 100168).

### 3.4. Chemicals and Positive Control

Hesperidin (Lot No. 090M1879V) and hesperetin (Lot No. 129K1367) were purchased from Sigma-Aldrich (St. Louis, MO, USA). Fluoxetine hydrochloride (Lot No. 2597489) was purchased from LKT Laboratories Inc. (St. Louis, MN, USA) as a positive control for the elevated open-platform test. 

### 3.5. Elevated Open-Platform Test

The anti-anxiety like effect was evaluated with the elevated open-platform test according to the method described by Miyata *et al.* [16]. Briefly, mice were placed on the bottom (open-platform) of upside-down glass beaker (11.0 cm diameter, 15.2 cm high), and the behavioral response to slipping off the platform, namely freezing behavior, defined as a state without movements except for respiration, was recorded as an index of anxiety by a video camera and an observer blindly measured the duration of freezing behavior within 10 min. 

The extracts and chemicals were suspended in the distilled water just before use and administered to mice orally at a dose of 100, 500 mg/kg (Chimpi extracts), 800 mg/kg (YKH extract), and 50 mg/kg (hesperidin and hesperetin). The dose of YKH was calculated on the basis of clinical dose (5,000 mg/day/adult), and Chimpi extracts and hesperidin were based on their ratio or contents in their formula or crude drugs, respectively. Fluoxetine hydrochloride was dissolved in saline and administrated intraperitoneally to mice at a dose of 10 mg/kg.

### 3.6. Locomotor Activity Test

Locomotor activity of mice was measured by using a Supermex^®^ apparatus (Muromachi Kikai Co., Ltd., Tokyo, Japan). The apparatus detects the movement of mice by means of a far-infrared-ray sensor and records it on a digital counter. The sensor detects the radiated body heat of an animal, and records all spontaneous movements, both vertical and horizontal, by counting the number of times a photocell beam is interrupted or blocked [23]. After 60 min of treatment with each sample, the mice treated with drugs were placed individually in empty plastic cages (16 × 26 × 13 cm), and then recording was started to measure automatically their movement for 10 min.

### 3.7. Statistical Analysis

The data are expressed as mean ± standard error of means (S.E.M). Significant differences were determined by one-way analysis of variance (ANOVA) followed by Dunnett’s test for multiple comparisons. P values of less than 0.05 were considered statistically significant.

## 4. Conclusions

In conclusion, Chimpi both from *C. unshiu* and *C. reticulata* possess significant antianxiety-like effects, and the major chemical constituent, hesperidin could be an active principle responsible for the antianxiety-like effects with a direct and/or indirect role via its aglycone hesperetin. Moreover, the results suggested that the possible pharmacological action of Chimpi against anxiety-like effects might be similar to that of fluoxetine, a selective inhibitor of serotonin reuptake, that is it could be associated with the serotonergic neurotransmission pathway. The results also provide evidence for the use of the YKH formula to treat behavioral and psychiatric symptoms of dementia (BPSD).
